# Semantic integration of clinical laboratory tests from electronic health records for deep phenotyping and biomarker discovery

**DOI:** 10.1038/s41746-019-0110-4

**Published:** 2019-05-02

**Authors:** Xingmin Aaron Zhang, Amy Yates, Nicole Vasilevsky, J. P. Gourdine, Tiffany J. Callahan, Leigh C. Carmody, Daniel Danis, Marcin P. Joachimiak, Vida Ravanmehr, Emily R. Pfaff, James Champion, Kimberly Robasky, Hao Xu, Karamarie Fecho, Nephi A. Walton, Richard L. Zhu, Justin Ramsdill, Christopher J. Mungall, Sebastian Köhler, Melissa A. Haendel, Clement J. McDonald, Daniel J. Vreeman, David B. Peden, Tellen D. Bennett, James A. Feinstein, Blake Martin, Adrianne L. Stefanski, Lawrence E. Hunter, Christopher G. Chute, Peter N. Robinson

**Affiliations:** 1 The Jackson Laboratory for Genomic Medicine, Farmington CT, 06032 USA; 2 Oregon Clinical and Translational Research Institute, Oregon Health and Science University, Portland, OR 97239 USA; 3 Department of Medical Informatics and Clinical Epidemiology, Oregon Health and Science University, Portland, OR 97239 USA; 4 Library, Oregon Health and Science University, Portland, OR 97239 USA; 5 Computational Bioscience Program, Department of Pharmacology, University of Colorado Anschutz School of Medicine, Aurora, CO 80045 USA; 6 Environmental Genomics and Systems Biology Division, Lawrence Berkeley National Laboratory, Berkeley, CA 94720 USA; 7 North Carolina Translational and Clinical Sciences Institute (NC TraCS), University of North Carolina at Chapel Hill, Chapel Hill, NC 27599 USA; 8 Genetics Department, University of North Carolina at Chapel Hill, Chapel Hill, NC 27599 USA; 9 School of Information and Library Sciences, University of North Carolina at Chapel Hill, Chapel Hill, NC 27599 USA; 10 Renaissance Computing Institute, University of North Carolina at Chapel Hill, Chapel Hill, NC 27599 USA; 11 Genomic Medicine Institute, Geisinger Health System, Danville, PA 17822 USA; 12 Institute for Clinical and Translational Research, Johns Hopkins University, Baltimore, MD 21202 USA; 13 Charité Centrum für Therapieforschung, Charité - Universitätsmedizin Berlin Corporate Member of Freie Universität Berlin, Humboldt-Universität zu Berlin, and Berlin Institute of Health, Berlin, 10117 Germany; 14 Einstein Center Digital Future, Berlin, 10117 Germany; 15 Linus Pauling Institute and Center for Genome Research and Biocomputing, Oregon State University, Corvallis, OR 97331 USA; 16 Lister Hill National Center for Biomedical Communications, National Library of Medicine, National Institutes of Health, Bethesda, MD 20894 USA; 17 Department of Medicine, Indiana University School of Medicine, Indianapolis, IN 46202 USA; 18 Center for Biomedical Informatics, Regenstrief Institute, Inc., Indianapolis, IN 46202 USA; 19 Division of Allergy, Immunology and Rheumatology, Department of Pediatrics, University of North Carolina, Chapel Hill, NC 27599 USA; 20 University of North Carolina Center for Environmental Medicine, Asthma and Lung Biology, University of North Carolina, Chapel Hill, NC 27599 USA; 21 Department of Pediatrics, Section of Pediatric Critical Care, University of Colorado School of Medicine, Aurora, CO 80045 USA; 22 Adult and Child Consortium for Health Outcomes Research and Delivery Science (ACCORDS), University of Colorado School of Medicine, Aurora, CO 80045 USA; 23 Institute for Systems Genomics, University of Connecticut, Farmington, CT 06032 USA

**Keywords:** Translational research, Asthma, Predictive markers

## Abstract

Electronic Health Record (EHR) systems typically define laboratory test results using the Laboratory Observation Identifier Names and Codes (LOINC) and can transmit them using Fast Healthcare Interoperability Resource (FHIR) standards. LOINC has not yet been semantically integrated with computational resources for phenotype analysis. Here, we provide a method for mapping LOINC-encoded laboratory test results transmitted in FHIR standards to Human Phenotype Ontology (HPO) terms. We annotated the medical implications of 2923 commonly used laboratory tests with HPO terms. Using these annotations, our software assesses laboratory test results and converts each result into an HPO term. We validated our approach with EHR data from 15,681 patients with respiratory complaints and identified known biomarkers for asthma. Finally, we provide a freely available SMART on FHIR application that can be used within EHR systems. Our approach allows readily available laboratory tests in EHR to be reused for deep phenotyping and exploits the hierarchical structure of HPO to integrate distinct tests that have comparable medical interpretations for association studies.

## Introduction

Electronic health records (EHRs) have been widely adopted in US hospitals since the Health Information Technology for Electronic and Clinical Health Act (HITECH) was passed in 2009, and offer an unprecedented opportunity to accelerate translational research because of advantages of scale and cost efficiency as compared with traditional cohort-based studies.^1^ In particular, EHRs contain rich phenotype information that can be utilized to stratify diseases and to develop hypotheses. For instance, phenome-wide association studies (PheWAS) can exploit EHR data to define case–control cohorts for disease diagnoses or laboratory traits and then analyze associations with hundreds of thousands of genetic variants.^2–4^ Despite the great potential of EHR data, patient phenotyping from EHRs is still challenging because the phenotype information is distributed in many EHR locations (laboratories, notes, problem lists, imaging data, etc.) and since EHRs have vastly different structures across sites. This lack of integration represents a substantial barrier to widespread use of EHR data in translational research.

Laboratory tests provide a critical resource for phenotype extraction. Deep phenotyping, i.e., comprehensive and precise phenotyping of individual disease manifestations, is an essential component of precision medicine and could potentially extend the reach of PheWAS studies.^5,6^ Laboratory tests have broad applicability for translational research, but EHR-based research using laboratory data have been challenging because of their diversity and the lack of standardization of reporting laboratory test results. For instance, some tests measure nitrite level in urine using an automated machine, whereas others use a test strip. Some report the value in mg/dL, whereas others report a qualitative value of positive/negative. If any of these tests were abnormal, the medical interpretation would be that nitrituria is present, yet current informatics frameworks do not easily support such inferences. Therefore, substantial challenges exist for standardization and integration of laboratory data for deep phenotyping and EHR-based translational research.

Recent advances in the standardization of EHR systems and phenotype ontologies make it feasible to extract patient phenotypes from laboratory tests at a large scale. The Fast Healthcare Interoperability Resource (FHIR) was introduced in 2013 and provides a standardized interface to individual EHR systems for healthcare-related data.^7^ FHIR separates healthcare-related data into granular components as “resources” such as observation, medication, patient identity and insurance claims, which have a standard definition and associated semantic bindings, which can be computationally integrated even when they are created by different methods and organizations. Laboratory tests, encoded as observations in FHIR, are uniquely identified with Laboratory Observation Identifier Names and Codes (LOINC), which is a universal code system that defines various kinds of clinical laboratory tests and other measurements (~86,000 entries).^8^ The outcome of a FHIR observation can be represented by a term in the Human Phenotype Ontology (HPO), which is a logically defined vocabulary for describing medically relevant abnormal phenotypes.^9^ The HPO has become the de facto standard for computational phenotype analysis in genomics and rare disease.^10–12^ The HPO currently contains 14,184 terms (February, 2019) including a comprehensive representation of laboratory abnormalities such as hyperglycemia, thrombocytopenia, nitrituria, and increased urine alpha-ketoglutarate concentration. Here, we present a computational method that semantically harmonizes FHIR, LOINC, and HPO. The software rolls up LOINC terms for tests whose outcomes are medically comparable into common categories and interprets the outcome as HPO terms, thereby automatically extracting detailed, deep phenotypic profiles of laboratory results for downstream studies.

## Results

### Overview of strategy

We present an approach to mapping the outcomes of laboratory tests as encoded in EHRs with LOINC terms for the tests and FHIR Observation resources representing the test results as HPO terms. A LOINC term by itself does not specify the outcome of a test. But if the outcome of a test (such as “high” or “low”) and the nature of the test are known, we can then infer the phenotypic abnormality. For example, LOINC 32710-6 “Nitrite [Presence] in Urine” together with the outcome “positive” implies the phenotypic abnormality Nitrituria (HP:0031812).

LOINC-coded laboratory tests can be grouped broadly into three categories, those with a quantitative outcome (Qn), an ordered categorical outcome (ordinal, or Ord) and an unordered categorical outcome (nominal, or Nom). A quantitative test for an analyte has a normal range, and there are three types of mappings depending on the result of the test: L (lower than normal), N (normal), and H (higher than normal). Take, for instance, a test for the concentration of potassium in the blood (LOINC:6298-4, Fig. 1a). If the result is high, our procedure infers the corresponding HPO term for Hyperkalemia (HP:0002153). Analogously, a low result is mapped to Hypokalemia (HP:0002900). The HPO is an ontology of abnormal phenotypes, and thus there is no term that specifically represents a normal test result. However, computational analysis can record negated HPO terms, and the normal test result is represented as NOT Abnormal blood potassium concentration (HP:0011042).

**Fig. 1:**
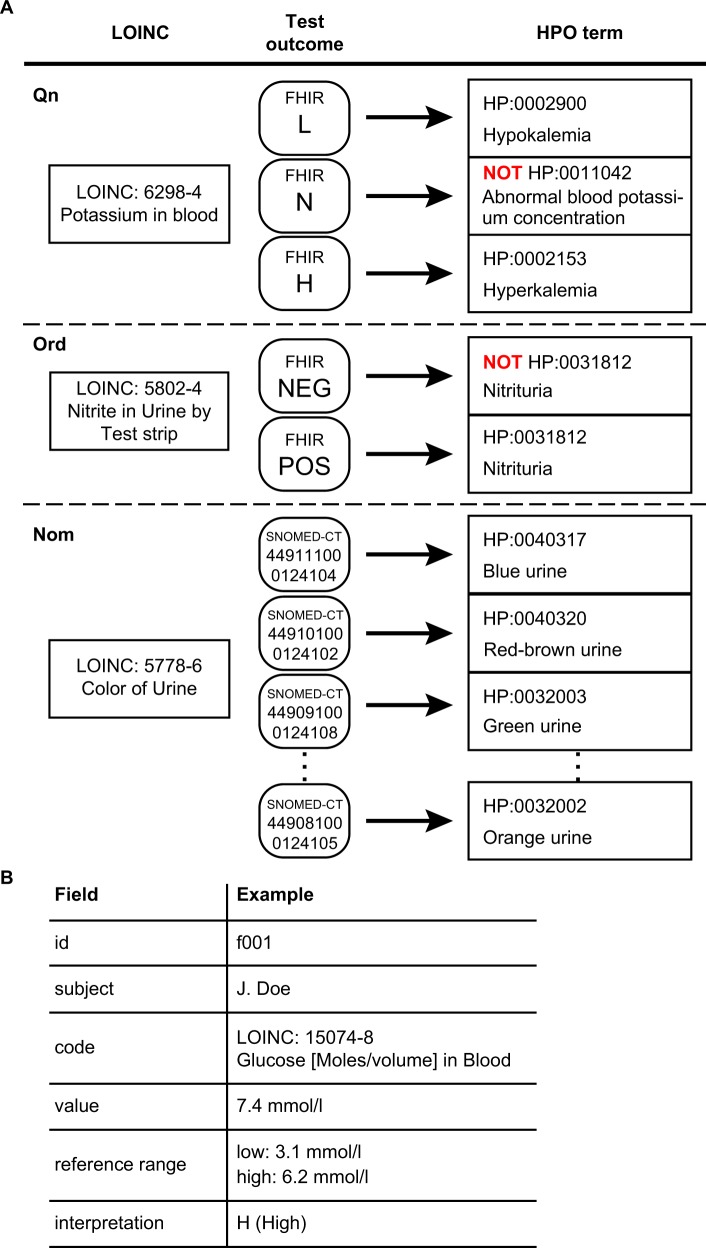
Semantic integration of LOINC-coded laboratory tests in FHIR into HPO terms. **a** Representative examples of LOINC to HPO mapping. Potassium in blood, a quantitative (Qn) LOINC test, has three potential outcomes, L (lower than normal), N (normal), and H (higher than normal) and is mapped to three corresponding HPO terms. Presence of nitrite in urine, an ordinal (Ord) test, has two possible outcomes, POS (positive) or NEG (negative) and is mapped to either Nitrituria (HP:0031812) or NOT Nitrituria (HP:0031812), respectively. Color of urine, a nominal (Nom) test, has a list of likely outcomes (represented as Systematized Nomenclature of Medicine-Clinical Terms [SNOMED-CT] codes using the SNOMED-CT US extension) and each one is mapped to an HPO term. **b** Schematic representation of the relevant contents of a FHIR observation for laboratory tests. Each FHIR Observation resource for a LOINC-encoded laboratory test includes an identifier (id) and the name of the patient, the LOINC code and name, the normal reference range and the observed value as well as an interpretation of the result (see Table 1 for a complete list)

Ordinal tests can have a series of ordered outcomes. The majority of the ordinal LOINC tests were mapped to two possible outcomes, POS (positive) or NEG (negative). For instance, the result of the test Nitrite in urine by test strip can be positive (present) or negative (absent) (Fig. 1a). If present, then our approach infers the HPO term Nitrituria (HP:0031812); if absent, our approach infers NOT Nitrituria (HP:0031812).

Nominal tests have a series of outcomes that lack a natural ordering. Yet, some nominal result values are considered abnormal. For instance, LOINC 5778-6, color of urine. Currently, nine abnormal results of this test are mapped to the nine child terms of abnormal urinary color (HP:0012086), including red urine (HP:0040318) and dark urine (HP:0040319).

### A LOINC to HPO mapping library

**Fig. 2:**
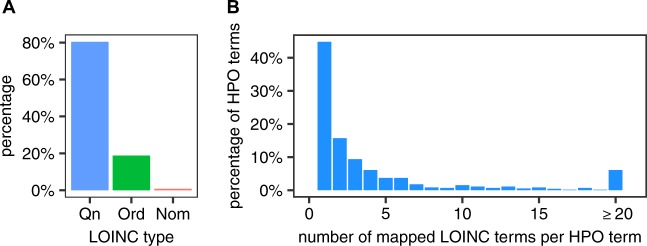
Quantification of the LOINC to HPO mapping library. **a** Distribution of annotated LOINC terms. **b** Distribution of HPO terms according to the number of LOINC terms mapped to an individual HPO term

We have mapped 2923 LOINC terms to HPO terms. In all, 80.4% of the mapped LOINC tests are Qn, 18.8% Ord, and 0.8% Nom (Fig. 2a). Taken together, these LOINC terms mapped to a total of 719 distinct HPO terms. We analyzed the distribution of the number of distinct LOINC terms that were mapped to an individual HPO term. In 54.8% of the cases, two or more LOINC terms are mapped to the same HPO term (mean 7.5) (Fig. 2b), reflecting the fact that multiple laboratory tests (and associated LOINC terms) have outcomes that we consider to have an equivalent clinical interpretation.

### Algorithm for converting LOINC-coded laboratory tests into HPO-coded phenotypes

We designed an algorithm that inspects elements of a FHIR resource for laboratory tests and converts the outcome into an HPO term. A standard FHIR resource for laboratory tests (a FHIR Observation) contains patient information, test identification, test result, normal reference ranges, and interpretations (Fig. 1b). The algorithm compares the numerical result with the normal reference ranges to assign an interpretation code such as “L” or “POS” (Table 1), or make use of the interpretation codes when they are present, to map the result to the corresponding HPO term (Supplementary Fig. 1). Overall, the algorithm handles all three major types of LOINC-coded laboratory tests (Qn, Ord, and Nom) when combined with the LOINC to HPO annotation data.

**Table 1:** FHIR codes for test outcomes

Primary code	Other FHIR codes mapped	Meaning
A	AA, W	Abnormal
L	<, D, LL, LU	Lower than normal
N	B, I	Normal
H	>, HH,HU, U	Higher than normal
NEG	ND, NR	Not present
POS	AC, DET, RR, TOX, WR	Present
U	HM, IE, IND, MS, NS, null, OBX, QCF, R, S, SDD, SYN-R, SYN-S, VS	Unknown

### HPO on FHIR

**Fig. 3:**
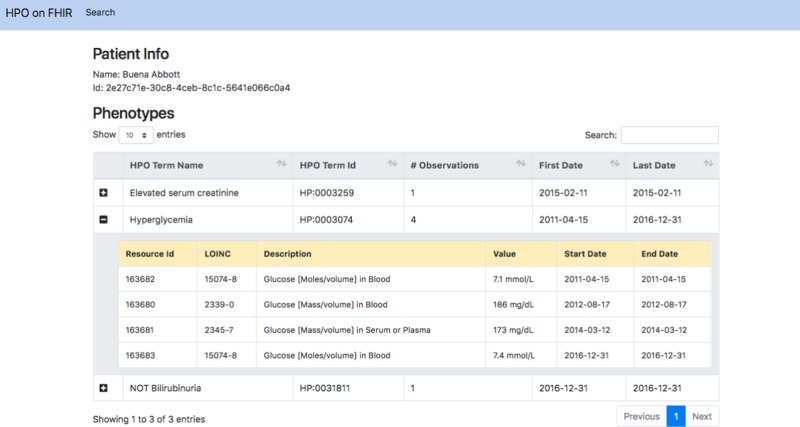
Screenshot of the HPO on FHIR application. We connected the HPO on FHIR application to the SmartHealthIT R3 Sandbox (a test server with synthetic data), queried all laboratory tests related to a simulated patient and converted all the laboratory tests into the corresponding HPO terms. The column “# Observations” shows the counts of laboratory tests that were mapped to the same HPO term, and for multiple tests mapped to the same HPO term, the dates of the first and last test are shown. In this example, LOINC 15074-8 was performed twice, and LOINC 2339-0 and 2345-7 were each performed once; the outcomes of all four tests were abnormally high (blood glucose), and so all four outcomes were mapped to the HPO term Hyperglycemia (HP:0003074)

To demonstrate conversion of FHIR-encoded LOINC tests into HPO, we created a SMART on FHIR application that uses the mapping library. SMART (Substitutable Medical Applications, Reusable Technologies) on FHIR is an application platform for EHRs that allows applications to run on different FHIR-enabled EHR systems.^13^ Our application, *HPO on FHIR*, transforms a bundle of laboratory observations for a patient into a list of HPO codes (Fig. 3). We have also developed a command-line application that can iterate through all laboratory tests in a FHIR-enabled server, convert each into an HPO term and store them in a relational database for translational research.

### LOINC to HPO demonstration with asthma

**Fig. 4:**
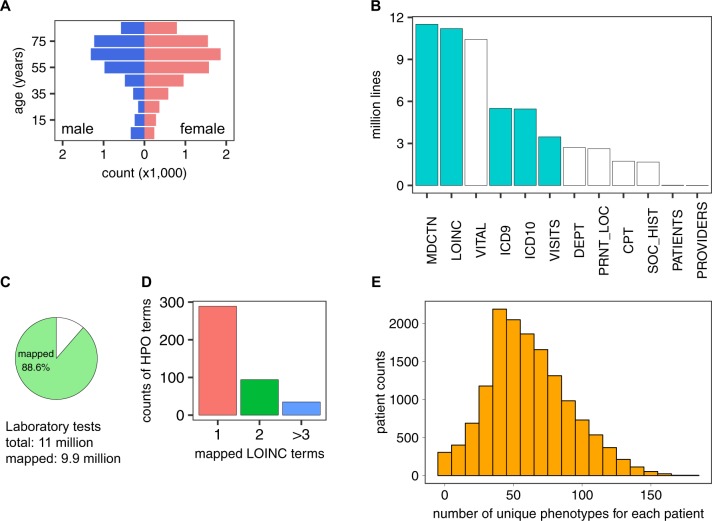
Analysis of UNC asthma dataset on asthma- and asthma-like patients. **a** Age and sex distribution of patients. **b** Categories of information extracted from the EHR data. Cyan, used for current research; white, not used for current research. MDCTN medication, LOINC LOINC-coded laboratory tests, VITAL vital signs, ICD 9 ICD 9-coded diagnosis, ICD 10 ICD 10-coded diagnosis, VISITS patient visit records, DEPT clinic location, PRNT_LOC hospital location, CPT CPT-coded procedures, SOC_HIST social history, PATIENTS patient identification records, PROVIDER provider information. **c** Percentage of laboratory tests in our dataset that could be converted into HPO terms (the remaining unmapped tests did not have LOINC to HPO annotations). **d** Number of LOINC terms mapped to a given HPO term in the UNC dataset. **e** Distribution of patients by the number of unique HPO terms that are mapped to each patient

To test our method for semantic integration of laboratory tests, we analyzed a de-identified EHR dataset from the University of North Carolina (UNC) comprising 15,681 patients who had a history of asthma or asthma-like symptoms. The cohort is skewed toward female (58.9%) and older patients (median age: 61.5 years, Fig. 4a). The median tracking period of patients in this cohort is 3.1 years. The dataset contains ~54 million records of LOINC-encoded clinical test results, medication prescriptions, diagnosis codes, procedure codes, patient information, and other supporting records (Fig. 4b). Using our LOINC to HPO conversion algorithm, we successfully transformed 9.9 out of 11 million (88.6%) laboratory tests into HPO terms (Fig. 4c). For the entire cohort, on average, each HPO term was mapped from 1.8 distinct types of laboratory tests (Fig. 4d), indicating that the transformation successfully integrated distinctly coded laboratory tests that have the same clinical interpretation. The mapping procedure assigned an average of 633 laboratory test-derived HPO terms per individual patient, many of which were from the same laboratory tests performed at different visits. The tests corresponded to a mean of 57.7 unique HPO terms, of which 20.8 were abnormalities and the remainder were normal phenotypes (Fig. 4e). The hierarchical structure of the HPO allows inferences to be propagated up to parent terms and their ancestors;^14^ using this method, we inferred an additional 51.2 HPO terms (total 73.5) based on 22.2 abnormalities for each patient (Supplementary Fig. 2).

As a proof-of-principle, we tested the ability of our procedure to identify phenotypic abnormalities associated with a diagnosis of asthma or with frequent prednisone use. About one-third of the patients in this cohort had an ICD 9/10 diagnosis of asthma, and the remaining patients had ICD 9/10 codes reflecting other, potentially asthma-like, respiratory complaints. In all, 14.2% of patients who had a diagnosis of asthma were administered or prescribed prednisone >3 times within a tracking period between 2004 and 2016; 8.5% of the remaining patients had been administered prednisone more than three times. Prednisone is a corticosteroid drug used for severe asthma treatment with multiple other indications.^15^ We reasoned that both the diagnosis of asthma and the history of treatment with prednisone would likely be correlated with different but overlapping sets of laboratory abnormalities. Using logistic regression, we assessed the contribution of frequent prednisone prescription and the presence of acute asthma diagnosis to each phenotypic abnormality.

Prednisone usage was significantly associated with an increased odds ratio for exhibiting many abnormal phenotypes that are consistent with the known effects of prednisone (Table 2), such as hypoalbuminemia (HP:0003073),^16^ neutrophilia (HP:0011897),^17^ monocytosis (HP:0012311),^18^ leukocytosis (HP:0001974),^18^ hypokalemia (HP:0002900),^19^ and elevated serum creatine phosphokinase (HP:0003236).^20^ An acute asthma diagnosis was significantly associated with seven phenotypes, abnormal metabolism (HP:0032245), abnormality of vitamin metabolism (HP:0100508), increased red blood cell count (HP:0020059), increased VLDL cholesterol concentration (HP:0003362), and eosinophilia (HP:0001880), and two ancestor terms of eosinophilia, abnormal eosinophil count (HP:0020064), and abnormal eosinophil morphology (HP:0001879). Eosinophilia is a well-established marker for acute allergic asthma.^21^ Several studies have linked vitamin A, B, C, D, E with asthma.^22–24^ In this study, we applied a threshold minimum number of patients before performing statistical analysis, and none of the specific subtypes of abnormality of vitamin metabolism (HP:0100508; *n* = 111 patients) passed this threshold. However, a number of patients were found to have increased blood folate (HP:0040087; *n* = 33 patients), vitamin B12 deficiency (HP:0200502; *n* = 6 patients), low serum calciferol (HP:0012053; *n* = 56 patients), and low serum calcitriol (HP:0012052; *n* = 6 patients). Thus, the hierarchical structure of HPO allowed us to infer the parent phenotype (Abnormality of vitamin metabolism) and aggregate enough data to find that it is associated with acute asthma diagnosis (Supplementary Fig. 3). The term abnormal metabolism (HP:0032245) was also flagged, but this was solely related to the 111 patients annotated to abnormality of vitamin metabolism, which is a child term of abnormal metabolism. Although there have been some conflicting results,^25^ a number of studies have shown a positive correlation between increased total, high- or low density lipoprotein cholesterol, or triglycerides (Supplementary Fig. 2) and asthma.^26–30^ An increased red blood cell count is not a recognized biomarker of asthma, but could conceivably reflect a number of factors including hypoxemia (11.1% with an acute asthma diagnosis also had a chronic obstructive pulmonary disease diagnosis), or hemoconcentration resulting from acute dehydration during an asthma attack, but the nature of this retrospective study does not allow us to consult the full medical records to investigate this.

**Table 2:** Odds ratio of phenotypes for frequent prednisone prescription and acute asthma diagnosis

HPO	Frequent prednisone prescription				Acute asthma diagnosis			
	Odds ratio	Confidence interval (95%)	*P-*value		Odds ratio	Confidence interval (95%)	*P*-value	
Abnormal metabolism	0.56	[0.26–1.23]	1.45 × 10^-1^	-	1.72	[1.16–2.55]	6.78 × 10^-3^	**
Abnormality of vitamin metabolism	0.56	[0.26–1.23]	1.45 × 10^-1^	-	1.72	[1.16–2.55]	6.78 × 10^-3^	**
Increased red blood cell count	2.48	[2–3.07]	5.42 × 10^-17^	**	1.5	[1.25–1.79]	9.24 × 10^-6^	**
Increased VLDL cholesterol concentration	0.77	[0.38–1.53]	4.47 × 10^-1^	-	1.49	[1–2.23]	4.84 × 10^-2^	*
Abnormal VLDL cholesterol concentration	0.72	[0.36–1.44]	3.50 × 10^-1^	-	1.42	[0.96–2.1]	7.91 × 10^-2^	-
Increased hematocrit	2.42	[1.89–3.11]	2.21 × 10^-12^	**	1.23	[0.99–1.53]	5.35 × 10^-2^	-
Abnormal eosinophil count	3.72	[3.17–4.37]	1.42 × 10^-59^	**	1.17	[1.01–1.36]	3.06 × 10^-2^	*
Abnormal eosinophil morphology	3.72	[3.17–4.37]	1.42 × 10^-59^	**	1.17	[1.01–1.36]	3.06 × 10^-2^	*
Eosinophilia	3.74	[3.19–4.39]	7.58 × 10^-60^	**	1.17	[1.01–1.36]	3.14 × 10^-2^	*
Reduced blood urea nitrogen	2.35	[2.01–2.76]	6.46 × 10^-27^	**	1.08	[0.95–1.24]	2.40 × 10^-1^	-
Increased LDL cholesterol concentration	0.81	[0.57–1.15]	2.28 × 10^-1^	-	1.07	[0.86–1.33]	5.39 × 10^-1^	-
Hypercholesterolemia	2.99	[2.58–3.47]	5.62 × 10^-48^	**	1.05	[0.93–1.19]	4.48 × 10^-1^	-
Abnormal LDL cholesterol concentration	0.85	[0.61–1.19]	3.33 × 10^-1^	-	1.02	[0.82–1.26]	8.71 × 10^-1^	-

**P < 0.01, *P < 0.05, ^-^*P* ≥ 0.05; table is sorted by the odds ratio for acute asthma diagnosis. Only HPO terms of which the odds ratio > 1 for acute asthma diagnosis are shown. Refer to Supplementary Table 3 for all terms

## Discussion

In this report, we present an approach to the semantic integration of laboratory tests and results in EHR data. Our approach connects a widely used system for denoting laboratory tests, LOINC, with a current standard for transmitting healthcare information, FHIR, and a computational resource for deep phenotyping, HPO, that was previously used mainly in the context of rare disease research and diagnostics. Previous work such as OntoServer provides lookup services of different terminologies and maps similar concepts that originate from different terminologies.^31^ The focus of our tool in contrast is to provide a means of interpreting the outcomes of laboratory tests using an ontology of phenotypic abnormalities. Normalizing laboratory tests with HPO terms is an effective solution for two fundamental issues in clinical research: data integration and deep phenotyping. Laboratory test results support a large proportion of medical decisions.^32^ It is common that different laboratory tests may lead to results that have very similar or identical clinical interpretations. These different tests are recorded in the EHR using distinct codes (for instance, currently, there are four different LOINC terms for different tests of urine nitrite). This level of granularity can create difficulties for the semantic integration of comparable test results. By converting the results of laboratory tests to HPO-encoded phenotypes, our method provides an effective way for integrating laboratory tests that have the same clinical interpretation but different LOINC codes. Extracted patient phenotypes can be directly utilized for PheWAS studies, which is important because phenotyping patients is a major bottleneck for conducting PheWAS studies.^33^ The Electronic Medical Records and Genomics (eMerge) network develops EHR-derived phenotyping algorithms by combining diagnosis codes, procedure codes, medication, narratives, and subsets of laboratory tests and iteratively refine them to identify control and disease cohorts for genome-wide association studies and PheWAS.^1,3,33–35^ Our method complements existing phenotyping algorithms because it extracts additional phenotypic information by systematically interrogating the vast amount of data in laboratory tests.

The analysis of UNC EHR data demonstrated the potential of combining deep phenotypes from our tool with EHR data for biomarker discovery. Our current mapping library allowed us to convert the majority of the laboratory tests into HPO terms and assign an average of 57.7 unique phenotypes to each patient. The statistical analysis identified phenotypic abnormalities that are associated with frequent prescriptions of prednisone and/or acute asthma diagnosis. The cohort used for this analysis is biased toward senior and female patients and may not be reflective of asthma patient distributions, but the fact that our analysis identified numerous abnormalities that are associated with either prednisone use or asthma suggests that our approach can be useful for the investigation of EHR data for laboratory-based biomarkers of diseases and conditions. We have demonstrated the utility of our approach on the UNC dataset using a simple logistic regression approach as a proof-of-principle; we envision that our mapping approach could be used together with a variety of statistical and algorithmic analysis strategies to address a variety of topics in EHR-based translational research, and we have therefore coded our foundational approach in a way that can easily be integrated into other statistical analysis pipelines. A particularly attractive direction is to incorporate temporal information to build predictive models based on longitudinal phenotypic timelines.^36,37^

Some practical issues need to be considered when adopting our approach. Although LOINC has been widely adopted by healthcare providers and increasingly mandated by various federal agencies, it is still not a universal system. Since we used LOINC for the mapping, locally coded laboratory tests will not be able to be mapped to HPO terms with our tools. Similarly, the SMART on FHIR tool reported here can only be utilized in FHIR-enabled hospital systems. However, our annotation file and the algorithmic approach we adopted can be used independently of FHIR.

Several other use cases for our approach are conceivable. Rule-based algorithms could be applied to infer HPO terms from the primary phenotypic abnormalities. For instance, the combination of decreased hemoglobin concentration (HP:0020062) and decreased mean corpuscular volume (HP:0025066) implies microcytic anemia (HP:0001935). The HPO is widely used in rare disease diagnostics, but one bottleneck is that in many settings, HPO terms need to be entered manually into the analysis software. A recent study used text-mining to extract detailed patient phenotypes through natural language processing of clinical narratives in EHR, and used the resulting lists of HPO terms for genomic diagnostics.^11^ Our tool could supplement such approaches by providing a computational representation of laboratory findings to genomic diagnostic software such as Exomiser.^38–40^ In principle, our tool could be used to support other tasks related to EHR data, including decision support and cohort recruitment. In the future, we anticipate that semantic integration of a wider range of EHR data will become the norm to support data-driven translational research and precision medicine.

## Methods

### Mapping LOINC terms to HPO terms

We performed manual biocuration to construct a mapping library from each potential outcome of a LOINC test to the corresponding HPO term (Fig. 1a). The test outcome is represented using a subset of FHIR codes (Table 1, primary code), such as “lower than normal”, “normal”, or “higher than normal”. For quantitative tests that report a numeric measurement, we use FHIR interpretation code “L” and “H” to indicate lower or higher than normal, and “N” and “A” to indicate the result is normal or abnormal. For ordinal tests that have a binary outcome, i.e., present or absent of the test target, we use FHIR interpretation code “POS” to indicate present and “NEG” to indicate absent. In addition, other interpretation codes defined by FHIR are first mapped to primary codes. For example, FHIR codes “LL” (critically low) and “<” (off scale low) are both mapped to “L” (Table 1).

The value for a map entry is an HPO term accompanied by a boolean value to indicate whether it should be negated. That is, while an abnormal test outcome is mapped to a particular HPO term, the normal outcome for that test is mapped to the negated form, since the HPO contains only terms for abnormal phenotypes. Figure 1a shows three examples of mappings for Qn, Ord, or Nom LOINC terms.

In order to efficiently perform the biocuration needed to generate the LOINC mappings, we developed a JavaFX-based annotation tool that recommends candidate HPO terms to a LOINC test based on lexical matching between HPO term definitions and the name of a laboratory test. The recommended HPO terms were then manually vetted by one of five biocurators (i.e., one MD and four PhDs who have biomedical training and are major contributors to the HPO project) and cross-validated by a different annotator. Mapping problems were tracked by Github issues (https://github.com/TheJacksonLaboratory/loinc2hpoAnnotation/issues) and discussed during regular meetings. Source code and an executable version of the biocuration application are freely accessible at https://github.com/monarch-initiative/loinc2hpo. In addition, a subset (*n* = 160) of pediatric-specific laboratory tests were independently validated by five domain experts (i.e., three pediatric clinicians, a PhD-level molecular biologist, and a master’s-level epidemiologist). To perform this validation, a Qualtrics survey was designed so that each question featured a laboratory test description and set of reasonable HPO concepts. The survey was completed by all experts between October and December (2019). After completion, any laboratory test mapping that did not meet agreement by at least one clinician and both the biologist/epidemiologist were re-evaluated with one clinician until consensus was reached. The pediatric terms were additionally vetted on the loinc2hpoAnnotation GitHub tracker by the entire team of biocurators.

### LOINC to HPO mapping file

The LOINC to HPO mapping file contains records of mapping from LOINC test outcomes to the corresponding HPO terms. The annotation data are serialized as a tab-separated value (TSV) file. Each line records the LOINC code, test outcome, the mapped HPO term, and whether the mapped term should be negated. The annotation file is deposited at Github and can be accessed at https://w3id.org/loinc2hpo/annotations. An excerpt is shown in Supplementary Table 1.

### HPO on FHIR

We created a SMART on FHIR application, HPO on FHIR, to query a FHIR-enabled EHR servers and return patient laboratory results with LOINC codes and their corresponding HPO terms. The web interface of the application aggregates identical HPO terms together for visualization and also allows users to display source laboratory tests including subject, LOINC code, FHIR resource id, effective time and the corresponding HPO term. The application was written in the Java language with the Spring framework. The application implements the LOINC to HPO conversion algorithm described in Supplementary Fig. 1. The application is deposited at Github and can be accessed at (https://github.com/OCTRI/poc-hpo-on-fhir).

### Command-line application for gathering FHIR server statistics

We created a command-line application that finds all laboratory tests for a patient on a FHIR server and attempts to convert them to HPO. The conversion results, both successes and failures, are stored in a relational database to aid in translational research. We ran the application on seven common FHIR sandboxes and gathered statistics about the LOINCs encountered, the rate of success in conversion, and the underlying causes of failure. The application was written in the Java language with the Spring framework. Source code, results, and a backup of the database, can be accessed at https://github.com/OCTRI/f2hstats.

### Analysis of UNC data on patients with asthma or an asthma-like condition

For the purposes of demonstrating the potential utility of our library, we examined a de-identified EHR dataset extracted from the Carolina Data Warehouse for Health (CDWH) at the UNC. The data were accessed under a fully executed Data Use Agreement between The Jackson Laboratory and UNC. The CDWH is UNC Health Care System’s (UNCHCS) enterprise data warehouse, and contains EHR data for all UNCHCS patients from 2004 through 2016. The sample used for this investigation contains 15,681 patients with one or more encounters at UNCHCS with an asthma or asthma-like diagnosis (Supplementary Table 2). The data were exported from the UNC EHR system as eight separate comma-separated value (CSV) files containing clinical observations in a variety of data domains, including demographics, encounter details, diagnoses, procedures, medications, vital signs, and LOINC-coded lab results. Prior to transmission from UNC, the dataset was de-identified according to the Safe Harbor method of the Health Insurance Portability and Accountability Act (HIPAA), and all dates were shifted ±50 days. The project methods and use of the de-identified EHR-derived dataset were reviewed by The Jackson Laboratory Institutional Review Board and confirmed to be compliant with relevant guidelines and regulations and approved for data access on 19 December 2017.

Using the extracted laboratory data, we converted each LOINC-coded test into an HPO term. We note, however, that not every laboratory test result was captured in the available dataset. For each patient, we combined test records mapped to the same HPO terms and recorded the counts of observations for each HPO term. Then, we inferred additional phenotypic abnormalities based on the hierarchical structure of HPO, i.e., if a patient was assigned with an HPO term, we infer that the patient also had phenotypic abnormalities encoded by parent and other ancestor terms (Supplementary Fig. 2). We reasoned that an isolated abnormal measurement might represent an artifact or might not be typical of the clinical course of the patient, and therefore used a threshold of three observations over the entire observation period in order to classify a patient with the corresponding HPO-encoded phenotypic abnormality. We classified a patient not having an HPO-encoded phenotypic abnormality only when the patient had never been assigned to the HPO term in question. Patient age was calculated from the last hospital visit date subtracting the birth date and is subject to an inaccuracy of ±50 days due to the deidentification procedure (see above). Patients who rarely visited hospitals were less likely to receive laboratory tests and thus had less phenotypes, so we excluded those who had medical encounters on <10 days. Patients received >3 prednisone prescriptions were considered frequent users.

### Statistics

We applied a logistic regression model to determine the weights of being a frequent prednisone user (values 0 or 1) and having an acute asthma diagnosis (values 0 or 1) in determining a patient having an HPO-encoded phenotype (values 0 or 1). We excluded HPO terms from analysis of which the majority (95%) of the cohort had universal values (all 0 or 1). The natural exponential of the weights ± 1.98 standard deviations were converted to the odd ratio and 95% confidence intervals for each variable.

Data cleaning, normalization, wrangling, and table joining were conducted by a combination of “tidyverse”, “RSQLite” packages in R, SQLite, and Java. Logistic regression was conducted with the “glm” package in R. All source code is deposited at Github and can be accessed through https://github.com/TheJacksonLaboratory/HUSHDataAnalysis.

### Reporting summary

Further information on research design is available in the Nature Research Reporting Summary linked to this article.

## Supplementary information

Supplementary Material

reporting summary

## Data Availability

The patient EHR dataset can be acquired from the UNC with Data Use Agreement.

## Supplementary information

**Supplementary Information** accompanies the paper on the *npj Digital Medicine* website (10.1038/s41746-019-0110-4).

## Code availability

Computer code used in this study is openly accessible with the links provided in the Methods section.
