# Supplementary material for: Semantic integration of clinical laboratory tests from electronic health records for deep phenotyping and biomarker discovery
Source: NPJ Digit Med. 2019 May 2;2:32. doi: 10.1038/s41746-019-0110-4 (PMC6527418; doi:10.1038/s41746-019-0110-4)
Supplement: Supplementary file 1 — Supplementary Material [file 41746_2019_110_MOESM1_ESM.docx]

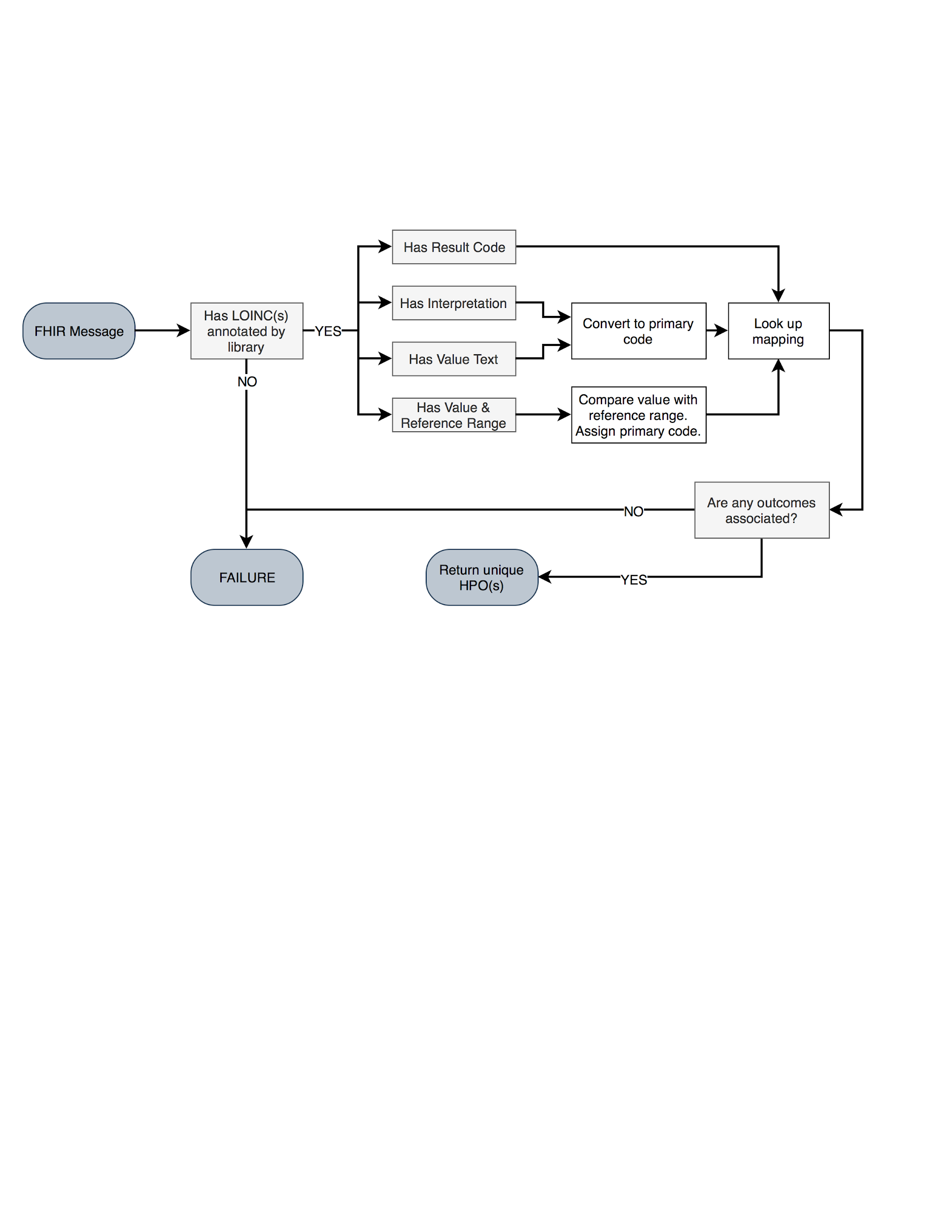


**Supplementary Figure 1**. FHIR to HPO conversion algorithm. Briefly, we extract the LOINC codes in the FHIR message, interpret the results with a code that is used in the mapping library, and then return the corresponding HPO term. If the result of a FHIR message is provided as a code used in the mapping library (such as the color of urine), the interpretation step is skipped; if the result of a FHIR message is provided with an interpretation code, the algorithm converts the interpretation code into a FHIR code used in Table 1; if the result is provided as a text string, the algorithm parses it and converts it to a FHIR code. For other tests, the algorithm compares the raw result with the reference ranges and assigns a code to represent the outcome. The algorithm was implemented as a Java library, fhir2hpo, with the Spring framework and can be accessed through the Github repository at <https://github.com/OCTRI/fhir2hpo>.

**
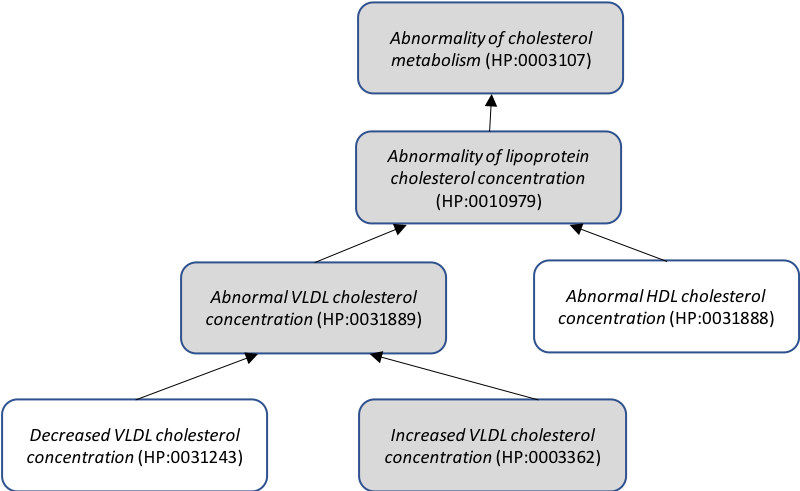
**

**Supplementary Figure 2.** Inference of phenotypic abnormalities with the hierarchy of HPO. The diagram demonstrates the tree-structured hierarchy with a few selected terms related to very low density lipoprotein (VLDL) cholesterol. The leaf terms contain more specific information than the parent term. We infer, for instance, that if a patient has the phenotype of *Increased VLDL cholesterol concentration* (HP:0003362), the patient also has abnormal phenotypes encoded by the ancestors of the current term, such as *Abnormal VLDL cholesterol concentration* (HP:0031889), *Abnormality of lipoprotein cholesterol concentration* (HP:0010979) and *Abnormality of cholesterol metabolism* (HP:0003107).


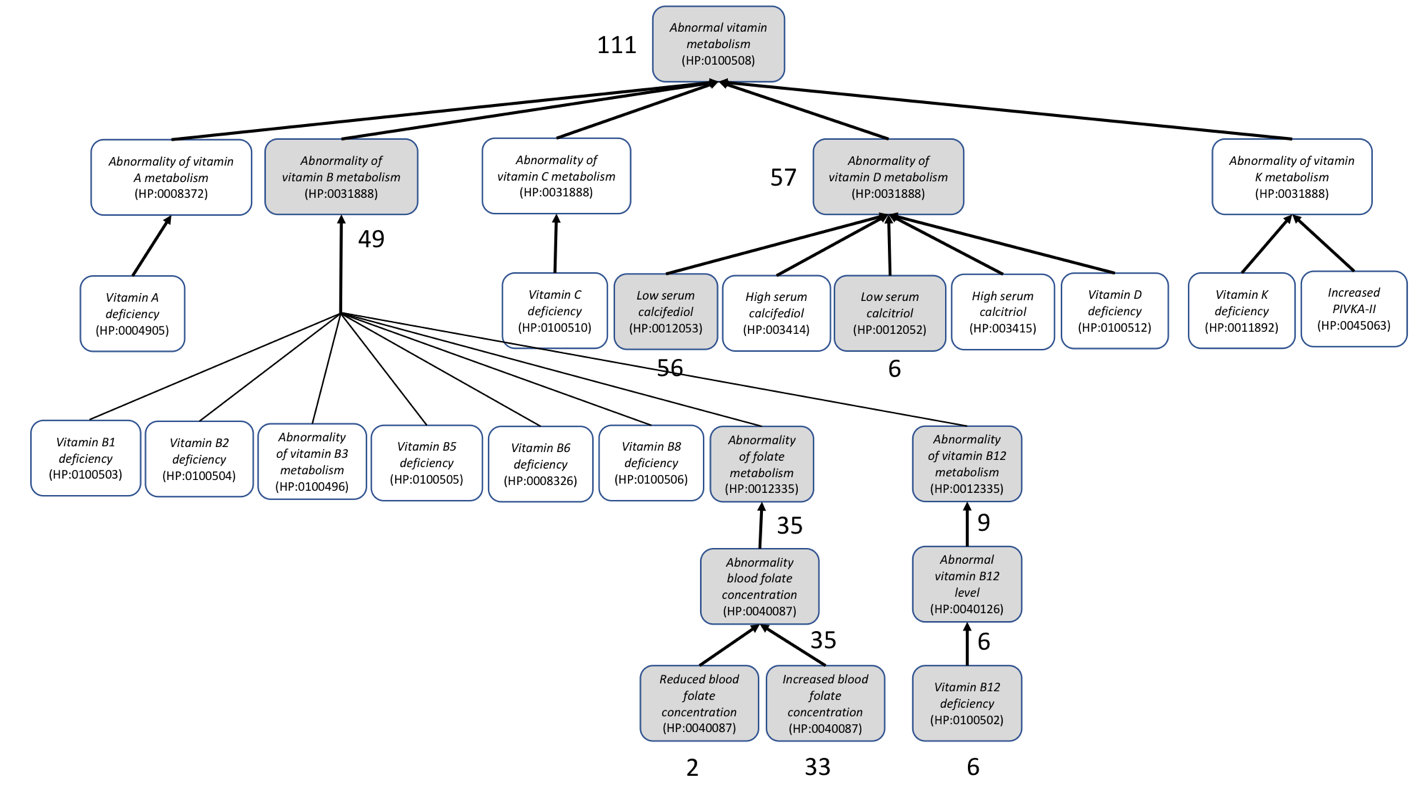


**Supplementary Figure 3.** Subontology of the HPO on abnormal vitamin metabolism. The number next to gray-colored terms indicates the count of patients that possessed the phenotype. For terms with no number, no patients were found with the phenotype in question.

**Supplementary Table 1**. Excerpt of LOINC to HPO annotation file

| **loincId** | **loincScale** | **system** | **code** | **hpoTermId** | **isNegated** |
| --- | --- | --- | --- | --- | --- |
| 38230-9 | Qn | FHIR | N | HP:0040077 | true |
| 38230-9 | Qn | FHIR | H | HP:0003072 | false |
| 38230-9 | Qn | FHIR | L | HP:0002901 | false |
| 5902-2 | Qn | FHIR | N | HP:0008151 | true |
| 5902-2 | Qn | FHIR | H | HP:0008151 | false |
| 777-3 | Qn | FHIR | N | HP:0011873 | true |
| 777-3 | Qn | FHIR | H | HP:0001894 | false |
| 777-3 | Qn | FHIR | L | HP:0001873 | false |
| 2019-8 | Qn | FHIR | H | HP:0012416 | false |
| 2019-8 | Qn | FHIR | L | HP:0012417 | false |
| 5803-2 | Qn | FHIR | L | HP:0031033 | false |
| ... | ... | ... | ... | ... | ... |

Columns from left to right: loincId, LOINC code; loincScale, type of the laboratory test; system and code, indicating the namespace and code for the test outcome; hpoTermId and isNegated, representing the mapped HPO and whether it needs to be negated for the outcome. Meta-data columns can be accessed online and are not shown here.

**Supplementary Table 2.** ICD codes used by UNC asthma dataset to identify asthma and asthma-like patients

| **Asthma diagnosis** | | **Asthma-like diagnosis** | |
| --- | --- | --- | --- |
| ICD-9: 493 | asthma | ICD-9:464 | croup |
| ICD-10: J45 | asthma | ICD-10:J05 | croup |
|  |  | ICD-9:496 | Reactive airway |
|  |  | ICD-10:J44 | Reactive airway |
|  |  | ICD-10:J66 | Reactive airway |
|  |  | ICD-9: 786 | cough |
|  |  | ICD-10: R05 | cough |
|  |  | ICD-9: 481-486 | pneumonia |
|  |  | ICD-10: J12-J18 | pneumonia |

Patients were collected into this dataset if they have one of the ICD codes.

**Supplementary Table 3**. Odds ratio of phenotypes for frequent prednisone prescription and acute asthma diagnosis

|  | **Frequent prednisone prescription** | | | | **Acute asthma diagnosis** | | | |
| --- | --- | --- | --- | --- | --- | --- | --- | --- |
| **HPO** | **Odds**  **ratio** | **Confidence Interval (95%)** | **P value** | | **Odds**  **ratio** | **Confidence Interval (95%)** | **P value** | |
| Abnormal metabolism | 0.56 | [0.26~1.23] | 1.45 x 10^-1^ | - | 1.72 | [1.16~2.55] | 6.78 x 10^-3^ | ** |
| Abnormality of vitamin metabolism | 0.56 | [0.26~1.23] | 1.45 x 10^-1^ | - | 1.72 | [1.16~2.55] | 6.78 x 10^-3^ | ** |
| Increased red blood cell count | 2.48 | [2~3.07] | 5.42 x 10^-17^ | ** | 1.5 | [1.25~1.79] | 9.24 x 10^-6^ | ** |
| Increased VLDL cholesterol concentration | 0.77 | [0.38~1.53] | 4.47 x 10^-1^ | - | 1.49 | [1~2.23] | 4.84 x 10^-2^ | * |
| Abnormal VLDL cholesterol concentration | 0.72 | [0.36~1.44] | 3.50 x 10^-1^ | - | 1.42 | [0.96~2.1] | 7.91 x 10^-2^ | - |
| Increased hematocrit | 2.42 | [1.89~3.11] | 2.21 x 10^-12^ | ** | 1.23 | [0.99~1.53] | 5.35 x 10^-2^ | - |
| Abnormal eosinophil count | 3.72 | [3.17~4.37] | 1.42 x 10^-59^ | ** | 1.17 | [1.01~1.36] | 3.06 x 10^-2^ | * |
| Abnormal eosinophil morphology | 3.72 | [3.17~4.37] | 1.42 x 10^-59^ | ** | 1.17 | [1.01~1.36] | 3.06 x 10^-2^ | * |
| Eosinophilia | 3.74 | [3.19~4.39] | 7.58 x 10^-60^ | ** | 1.17 | [1.01~1.36] | 3.14 x 10^-2^ | * |
| Reduced blood urea nitrogen | 2.35 | [2.01~2.76] | 6.46 x 10^-27^ | ** | 1.08 | [0.95~1.24] | 2.40 x 10^-1^ | - |
| Increased LDL cholesterol concentration | 0.81 | [0.57~1.15] | 2.28 x 10^-1^ | - | 1.07 | [0.86~1.33] | 5.39 x 10^-1^ | - |
| Hypercholesterolemia | 2.99 | [2.58~3.47] | 5.62 x 10^-48^ | ** | 1.05 | [0.93~1.19] | 4.48 x 10^-1^ | - |
| Abnormal LDL cholesterol concentration | 0.85 | [0.61~1.19] | 3.33 x 10^-1^ | - | 1.02 | [0.82~1.26] | 8.71 x 10^-1^ | - |
| All | 1 | [0~Inf] | 1.00 | - | 1 | [0~Inf] | 1.00 | - |
| Phenotypic abnormality | 1 | [0~Inf] | 1.00 | - | 1 | [0~Inf] | 1.00 | - |
| Leukocytosis | 4.54 | [3.96~5.2] | 1.59 x 10^-107^ | ** | 0.98 | [0.89~1.07] | 6.07 x 10^-1^ | - |
| Thrombocytosis | 2.56 | [2.19~3] | 6.24 x 10^-32^ | ** | 0.98 | [0.85~1.12] | 7.27 x 10^-1^ | - |
| Decreased mean platelet volume | 4.53 | [3.98~5.16] | 1.41 x 10^-118^ | ** | 0.97 | [0.87~1.07] | 5.05 x 10^-1^ | - |
| Abnormal myeloid leukocyte morphology | 4.97 | [4.26~5.79] | 7.09 x 10^-94^ | ** | 0.96 | [0.88~1.06] | 4.37 x 10^-1^ | - |
| Abnormal granulocyte count | 5.74 | [4.93~6.68] | 2.23 x 10^-115^ | ** | 0.94 | [0.85~1.03] | 1.59 x 10^-1^ | - |
| Abnormal granulocyte morphology | 5.74 | [4.93~6.68] | 2.23 x 10^-115^ | ** | 0.94 | [0.85~1.03] | 1.59 x 10^-1^ | - |
| Decreased mean corpuscular hemoglobin concentration | 2.67 | [2.33~3.06] | 2.56 x 10^-46^ | ** | 0.94 | [0.84~1.06] | 3.22 x 10^-1^ | - |
| Elevated hemoglobin A1c | 2.05 | [1.79~2.36] | 3.45 x 10^-25^ | ** | 0.93 | [0.83~1.03] | 1.65 x 10^-1^ | - |
| Abnormal circulating thyroxine level | 3.69 | [2.94~4.61] | 1.25 x 10^-30^ | ** | 0.92 | [0.74~1.14] | 4.37 x 10^-1^ | - |
| Abnormal erythrocyte sedimentation rate | 9.15 | [7.52~11.13] | 6.86 x 10^-111^ | ** | 0.92 | [0.75~1.14] | 4.54 x 10^-1^ | - |
| Abnormal hemoglobin | 2.18 | [1.9~2.5] | 1.33 x 10^-29^ | ** | 0.92 | [0.83~1.03] | 1.47 x 10^-1^ | - |
| Abnormal liver morphology | 2.84 | [2.5~3.22] | 3.38 x 10^-60^ | ** | 0.92 | [0.83~1.01] | 8.77 x 10^-2^ | - |
| Abnormal thyroid hormone level | 3.69 | [2.94~4.61] | 1.25 x 10^-30^ | ** | 0.92 | [0.74~1.14] | 4.37 x 10^-1^ | - |
| Abnormality of the abdominal organs | 2.84 | [2.5~3.22] | 3.38 x 10^-60^ | ** | 0.92 | [0.83~1.01] | 8.77 x 10^-2^ | - |
| Abnormality of the digestive system | 2.84 | [2.5~3.22] | 3.38 x 10^-60^ | ** | 0.92 | [0.83~1.01] | 8.77 x 10^-2^ | - |
| Abnormality of the liver | 2.84 | [2.5~3.22] | 3.38 x 10^-60^ | ** | 0.92 | [0.83~1.01] | 8.77 x 10^-2^ | - |
| Abnormality of the thyroid gland | 3.69 | [2.94~4.61] | 1.25 x 10^-30^ | ** | 0.92 | [0.74~1.14] | 4.37 x 10^-1^ | - |
| Abnormality of thyroid physiology | 3.69 | [2.94~4.61] | 1.25 x 10^-30^ | ** | 0.92 | [0.74~1.14] | 4.37 x 10^-1^ | - |
| Elevated erythrocyte sedimentation rate | 9.15 | [7.52~11.13] | 6.86 x 10^-111^ | ** | 0.92 | [0.75~1.14] | 4.54 x 10^-1^ | - |
| Elevated hepatic transaminase | 2.84 | [2.5~3.22] | 3.38 x 10^-60^ | ** | 0.92 | [0.83~1.01] | 8.77 x 10^-2^ | - |
| Elevated serum alanine aminotransferase | 2.89 | [2.52~3.32] | 5.34 x 10^-52^ | ** | 0.92 | [0.82~1.04] | 1.87 x 10^-1^ | - |
| Hyperlipidemia | 2.44 | [2.08~2.86] | 2.22 x 10^-28^ | ** | 0.92 | [0.81~1.05] | 2.24 x 10^-1^ | - |
| Hypertriglyceridemia | 2.44 | [2.08~2.86] | 2.22 x 10^-28^ | ** | 0.92 | [0.81~1.05] | 2.24 x 10^-1^ | - |
| Abnormal platelet volume | 6.15 | [5.33~7.1] | 1.72 x 10^-138^ | ** | 0.9 | [0.82~0.99] | 2.66 x 10^-2^ | * |
| Abnormality of the immune system | 5.38 | [4.37~6.61] | 1.06 x 10^-58^ | ** | 0.9 | [0.82~0.99] | 3.06 x 10^-2^ | * |
| Elevated C-reactive protein level | 7.84 | [6.56~9.38] | 5.72 x 10^-115^ | ** | 0.9 | [0.74~1.09] | 2.65 x 10^-1^ | - |
| Hematuria | 4.68 | [3.89~5.64] | 2.21 x 10^-60^ | ** | 0.9 | [0.75~1.09] | 2.77 x 10^-1^ | - |
| Hypoxemia | 3.09 | [2.58~3.7] | 2.30 x 10^-35^ | ** | 0.89 | [0.75~1.05] | 1.75 x 10^-1^ | - |
| Neutrophilia | 4.8 | [4.19~5.49] | 2.35 x 10^-116^ | ** | 0.89 | [0.81~0.99] | 2.41 x 10^-2^ | * |
| Abnormal cellular immune system morphology | 5.13 | [4.2~6.27] | 4.04 x 10^-59^ | ** | 0.88 | [0.8~0.96] | 5.76 x 10^-3^ | ** |
| Abnormal immune system morphology | 5.13 | [4.2~6.27] | 4.04 x 10^-59^ | ** | 0.88 | [0.8~0.96] | 5.76 x 10^-3^ | ** |
| Abnormal leukocyte count | 5.13 | [4.2~6.27] | 4.04 x 10^-59^ | ** | 0.88 | [0.8~0.96] | 5.76 x 10^-3^ | ** |
| Abnormal leukocyte morphology | 5.13 | [4.2~6.27] | 4.04 x 10^-59^ | ** | 0.88 | [0.8~0.96] | 5.76 x 10^-3^ | ** |
| Abnormality of urine homeostasis | 3.43 | [3.01~3.91] | 3.32 x 10^-77^ | ** | 0.88 | [0.79~0.97] | 7.25 x 10^-3^ | ** |
| Hypokalemia | 2.82 | [2.47~3.21] | 8.33 x 10^-56^ | ** | 0.88 | [0.79~0.98] | 1.40 x 10^-2^ | * |
| Acidemia | 2.92 | [2.45~3.48] | 1.23 x 10^-33^ | ** | 0.87 | [0.74~1.03] | 9.90 x 10^-2^ | - |
| Decreased serum iron | 3.84 | [3.17~4.65] | 4.58 x 10^-44^ | ** | 0.87 | [0.72~1.05] | 1.43 x 10^-1^ | - |
| Elevated serum anion gap | 3.84 | [3.3~4.47] | 1.93 x 10^-68^ | ** | 0.87 | [0.76~1] | 5.11 x 10^-2^ | - |
| Abnormal mean corpuscular hemoglobin concentration | 2.66 | [2.35~3.02] | 4.08 x 10^-53^ | ** | 0.86 | [0.78~0.96] | 5.39 x 10^-3^ | ** |
| Abnormal neutrophil count | 5.66 | [4.89~6.54] | 2.36 x 10^-124^ | ** | 0.86 | [0.79~0.95] | 2.28 x 10^-3^ | ** |
| Abnormality of neutrophils | 5.66 | [4.89~6.54] | 2.36 x 10^-124^ | ** | 0.86 | [0.79~0.95] | 2.28 x 10^-3^ | ** |
| Decreased serum creatinine | 1.69 | [1.48~1.94] | 1.73 x 10^-14^ | ** | 0.86 | [0.78~0.96] | 6.36 x 10^-3^ | ** |
| Hyperlipoproteinemia | 1.84 | [1.58~2.15] | 3.45 x 10^-15^ | ** | 0.86 | [0.76~0.97] | 1.12 x 10^-2^ | * |
| Abnormal circulating hormone level | 3.69 | [3.03~4.49] | 5.50 x 10^-40^ | ** | 0.85 | [0.7~1.03] | 8.66 x 10^-2^ | - |
| Abnormal urine cytology | 3.45 | [3.02~3.93] | 2.05 x 10^-78^ | ** | 0.85 | [0.77~0.94] | 2.13 x 10^-3^ | ** |
| Abnormality of brain morphology | 4.95 | [4.17~5.88] | 2.61 x 10^-76^ | ** | 0.85 | [0.72~1.01] | 5.73 x 10^-2^ | - |
| Abnormality of the hypothalamus-pituitary axis | 4.95 | [4.17~5.88] | 2.61 x 10^-76^ | ** | 0.85 | [0.72~1.01] | 5.73 x 10^-2^ | - |
| Abnormality of the pituitary gland | 4.95 | [4.17~5.88] | 2.61 x 10^-76^ | ** | 0.85 | [0.72~1.01] | 5.73 x 10^-2^ | - |
| Monocytosis | 2.04 | [1.76~2.38] | 1.33 x 10^-20^ | ** | 0.85 | [0.75~0.96] | 1.08 x 10^-2^ | * |
| Abnormal serum iron concentration | 4.25 | [3.55~5.07] | 5.89 x 10^-58^ | ** | 0.84 | [0.71~1.01] | 5.64 x 10^-2^ | - |
| Abnormality of blood and blood-forming tissues | 4.02 | [2.77~5.84] | 1.32 x 10^-13^ | ** | 0.84 | [0.72~0.98] | 2.08 x 10^-2^ | * |
| Elevated serum aspartate aminotransferase | 3 | [2.62~3.43] | 2.06 x 10^-58^ | ** | 0.84 | [0.75~0.94] | 1.78 x 10^-3^ | ** |
| Elevated serum creatine kinase | 2.88 | [2.4~3.45] | 9.50 x 10^-31^ | ** | 0.84 | [0.71~0.99] | 3.70 x 10^-2^ | * |
| Pyuria | 3.48 | [3~4.04] | 2.03 x 10^-62^ | ** | 0.84 | [0.74~0.96] | 9.65 x 10^-3^ | ** |
| Abnormal thyroid-stimulating hormone level | 5.08 | [4.27~6.04] | 7.65 x 10^-77^ | ** | 0.83 | [0.7~0.99] | 3.51 x 10^-2^ | * |
| Hypercapnia | 2.82 | [2.47~3.22] | 1.11 x 10^-53^ | ** | 0.83 | [0.74~0.92] | 4.94 x 10^-4^ | ** |
| Increased circulating thyroxine level | 3.69 | [2.84~4.8] | 5.11 x 10^-23^ | ** | 0.83 | [0.64~1.08] | 1.70 x 10^-1^ | - |
| Abnormal erythrocyte morphology | 2.74 | [2.22~3.37] | 1.81 x 10^-21^ | ** | 0.82 | [0.74~0.92] | 2.94 x 10^-4^ | ** |
| Abnormality of alkaline phosphatase activity | 2.41 | [2.1~2.76] | 1.00 x 10^-37^ | ** | 0.82 | [0.74~0.92] | 6.24 x 10^-4^ | ** |
| Abnormality of nervous system morphology | 5 | [4.24~5.9] | 1.48 x 10^-83^ | ** | 0.82 | [0.7~0.97] | 1.73 x 10^-2^ | * |
| Abnormality of the endocrine system | 5.67 | [4.94~6.52] | 4.83 x 10^-135^ | ** | 0.82 | [0.72~0.93] | 2.35 x 10^-3^ | ** |
| Abnormality of the genitourinary system | 3.92 | [3.41~4.5] | 4.13 x 10^-85^ | ** | 0.82 | [0.75~0.89] | 7.41 x 10^-6^ | ** |
| Abnormality of the nervous system | 5 | [4.24~5.9] | 1.48 x 10^-83^ | ** | 0.82 | [0.7~0.97] | 1.73 x 10^-2^ | * |
| Abnormality of the urinary system | 3.92 | [3.41~4.5] | 4.13 x 10^-85^ | ** | 0.82 | [0.75~0.89] | 7.41 x 10^-6^ | ** |
| Abnormality of the urinary system physiology | 3.92 | [3.41~4.5] | 4.13 x 10^-85^ | ** | 0.82 | [0.75~0.89] | 7.41 x 10^-6^ | ** |
| Morphological abnormality of the central nervous system | 5 | [4.24~5.9] | 1.48 x 10^-83^ | ** | 0.82 | [0.7~0.97] | 1.73 x 10^-2^ | * |
| Abnormal enzyme/coenzyme activity | 3.34 | [2.94~3.79] | 8.90 x 10^-79^ | ** | 0.81 | [0.73~0.89] | 3.65 x 10^-5^ | ** |
| Abnormal blood potassium concentration | 3.6 | [3.16~4.11] | 2.94 x 10^-83^ | ** | 0.8 | [0.73~0.88] | 4.07 x 10^-6^ | ** |
| Abnormal circulating cholesterol concentration | 2.48 | [2.17~2.83] | 1.60 x 10^-42^ | ** | 0.79 | [0.72~0.86] | 1.97 x 10^-7^ | ** |
| Hypernatremia | 3.23 | [2.7~3.86] | 1.25 x 10^-38^ | ** | 0.79 | [0.67~0.93] | 4.18 x 10^-3^ | ** |
| Abnormal blood carbon dioxide level | 3.98 | [3.48~4.55] | 1.07 x 10^-92^ | ** | 0.78 | [0.71~0.86] | 1.71 x 10^-7^ | ** |
| Abnormal blood oxygen level | 2.85 | [2.46~3.3] | 4.22 x 10^-46^ | ** | 0.78 | [0.68~0.88] | 1.06 x 10^-4^ | ** |
| Abnormal circulating creatine kinase concentration | 3.04 | [2.56~3.61] | 8.34 x 10^-38^ | ** | 0.78 | [0.66~0.92] | 2.37 x 10^-3^ | ** |
| Abnormal mean corpuscular volume | 2.34 | [2.06~2.65] | 1.25 x 10^-41^ | ** | 0.78 | [0.7~0.86] | 7.10 x 10^-7^ | ** |
| Abnormal renal physiology | 4.03 | [3.55~4.58] | 7.50 x 10^-104^ | ** | 0.78 | [0.7~0.85] | 1.63 x 10^-7^ | ** |
| Abnormal serum anion gap | 6.55 | [5.65~7.59] | 1.18 x 10^-140^ | ** | 0.78 | [0.71~0.86] | 1.30 x 10^-7^ | ** |
| Abnormality of acid-base homeostasis | 5.69 | [4.9~6.61] | 6.17 x 10^-118^ | ** | 0.78 | [0.71~0.85] | 2.71 x 10^-8^ | ** |
| Abnormality of circulating enzyme level | 3.04 | [2.56~3.61] | 8.34 x 10^-38^ | ** | 0.78 | [0.66~0.92] | 2.37 x 10^-3^ | ** |
| Abnormality of the kidney | 4.03 | [3.55~4.58] | 7.50 x 10^-104^ | ** | 0.78 | [0.7~0.85] | 1.63 x 10^-7^ | ** |
| Abnormality of the upper urinary tract | 4.03 | [3.55~4.58] | 7.50 x 10^-104^ | ** | 0.78 | [0.7~0.85] | 1.63 x 10^-7^ | ** |
| Acidosis | 3.19 | [2.68~3.79] | 9.74 x 10^-40^ | ** | 0.78 | [0.66~0.92] | 3.43 x 10^-3^ | ** |
| Elevated alkaline phosphatase | 2.41 | [2.09~2.77] | 9.34 x 10^-35^ | ** | 0.78 | [0.69~0.88] | 6.42 x 10^-5^ | ** |
| Hyperglycemia | 1.04 | [0.92~1.18] | 5.10 x 10^-1^ | - | 0.78 | [0.72~0.85] | 2.37 x 10^-8^ | ** |
| Increased HDL cholesterol concentration | 2.65 | [2.23~3.15] | 5.04 x 10^-29^ | ** | 0.78 | [0.67~0.91] | 1.82 x 10^-3^ | ** |
| Increased serum lactate | 3.19 | [2.68~3.79] | 9.74 x 10^-40^ | ** | 0.78 | [0.66~0.92] | 3.43 x 10^-3^ | ** |
| Abnormal blood gas level | 3.93 | [3.43~4.49] | 1.54 x 10^-90^ | ** | 0.77 | [0.71~0.85] | 3.16 x 10^-8^ | ** |
| Abnormal blood glucose concentration | 1.12 | [0.98~1.27] | 8.17 x 10^-2^ | - | 0.77 | [0.7~0.84] | 1.89 x 10^-9^ | ** |
| Abnormal circulating lipid concentration | 2.43 | [2.13~2.78] | 5.16 x 10^-40^ | ** | 0.77 | [0.71~0.84] | 2.79 x 10^-9^ | ** |
| Abnormal glucose homeostasis | 1.12 | [0.99~1.27] | 7.26 x 10^-2^ | - | 0.77 | [0.71~0.84] | 4.61 x 10^-9^ | ** |
| Abnormal monocyte count | 2.96 | [2.59~3.39] | 6.45 x 10^-57^ | ** | 0.77 | [0.69~0.87] | 9.06 x 10^-6^ | ** |
| Abnormality monocyte morphology | 2.96 | [2.59~3.39] | 6.45 x 10^-57^ | ** | 0.77 | [0.69~0.87] | 9.06 x 10^-6^ | ** |
| Abnormality of iron homeostasis | 3.89 | [3.34~4.54] | 5.51 x 10^-69^ | ** | 0.77 | [0.66~0.89] | 3.71 x 10^-4^ | ** |
| Abnormality of the respiratory system | 3.93 | [3.43~4.49] | 1.54 x 10^-90^ | ** | 0.77 | [0.71~0.85] | 3.16 x 10^-8^ | ** |
| Functional respiratory abnormality | 3.93 | [3.43~4.49] | 1.54 x 10^-90^ | ** | 0.77 | [0.71~0.85] | 3.16 x 10^-8^ | ** |
| Hyperproteinemia | 2.82 | [2.28~3.48] | 2.29 x 10^-22^ | ** | 0.77 | [0.63~0.94] | 1.02 x 10^-2^ | * |
| Hypochloremia | 2.56 | [2.25~2.91] | 2.69 x 10^-47^ | ** | 0.77 | [0.69~0.85] | 7.15 x 10^-7^ | ** |
| Abnormal blood ion concentration | 3.83 | [3.15~4.64] | 7.99 x 10^-43^ | ** | 0.76 | [0.69~0.84] | 2.73 x 10^-8^ | ** |
| Abnormal blood monovalent inorganic cation concentration | 3.6 | [3.13~4.15] | 3.17 x 10^-71^ | ** | 0.76 | [0.7~0.83] | 2.09 x 10^-9^ | ** |
| Abnormal blood transition element cation concentration | 3.91 | [3.36~4.56] | 2.94 x 10^-70^ | ** | 0.76 | [0.66~0.88] | 1.82 x 10^-4^ | ** |
| Abnormal thrombocyte morphology | 4.7 | [4.03~5.49] | 2.62 x 10^-87^ | ** | 0.76 | [0.69~0.83] | 1.22 x 10^-9^ | ** |
| Hyperoxemia | 2.84 | [2.4~3.36] | 1.55 x 10^-34^ | ** | 0.76 | [0.65~0.89] | 6.28 x 10^-4^ | ** |
| Increased RBC distribution width | 3.09 | [2.73~3.5] | 1.14 x 10^-72^ | ** | 0.76 | [0.7~0.83] | 7.42 x 10^-10^ | ** |
| Abnormal blood cation concentration | 3.39 | [2.88~4] | 5.15 x 10^-49^ | ** | 0.75 | [0.69~0.82] | 3.79 x 10^-10^ | ** |
| Abnormality of lipoprotein cholesterol concentration | 2.3 | [2.01~2.63] | 1.26 x 10^-34^ | ** | 0.75 | [0.68~0.83] | 1.25 x 10^-8^ | ** |
| Hypercalcemia | 2.79 | [2.25~3.46] | 2.82 x 10^-21^ | ** | 0.75 | [0.61~0.92] | 5.66 x 10^-3^ | ** |
| Hyperphosphatemia | 6.34 | [5.55~7.24] | 1.49 x 10^-166^ | ** | 0.75 | [0.67~0.85] | 5.07 x 10^-6^ | ** |
| Abnormal hemoglobin concentration | 3.14 | [2.73~3.63] | 7.40 x 10^-57^ | ** | 0.74 | [0.68~0.81] | 5.67 x 10^-12^ | ** |
| Hypocapnia | 3.49 | [3.06~3.98] | 2.85 x 10^-80^ | ** | 0.74 | [0.67~0.83] | 1.17 x 10^-7^ | ** |
| Abnormal blood phosphate concentration | 5.75 | [5.05~6.55] | 1.43 x 10^-155^ | ** | 0.73 | [0.65~0.82] | 3.78 x 10^-8^ | ** |
| Abnormal glomerular filtration rate | 3.71 | [3.29~4.2] | 2.06 x 10^-99^ | ** | 0.73 | [0.66~0.8] | 1.35 x 10^-10^ | ** |
| Abnormal hematocrit | 3.99 | [3.41~4.68] | 1.87 x 10^-67^ | ** | 0.73 | [0.66~0.79] | 6.82 x 10^-13^ | ** |
| Abnormal serum bicarbonate concentration | 3 | [2.58~3.5] | 9.32 x 10^-46^ | ** | 0.73 | [0.64~0.85] | 1.40 x 10^-5^ | ** |
| Decreased HDL cholesterol concentration | 1.98 | [1.67~2.36] | 5.17 x 10^-15^ | ** | 0.73 | [0.63~0.85] | 3.57 x 10^-5^ | ** |
| Decreased hemoglobin concentration | 2.99 | [2.61~3.42] | 1.37 x 10^-56^ | ** | 0.73 | [0.67~0.79] | 1.44 x 10^-13^ | ** |
| Decreased serum anion gap | 5.91 | [5.15~6.78] | 4.87 x 10^-145^ | ** | 0.73 | [0.67~0.81] | 2.05 x 10^-10^ | ** |
| Hypolipoproteinemia | 1.98 | [1.67~2.34] | 7.19 x 10^-16^ | ** | 0.73 | [0.63~0.84] | 8.91 x 10^-6^ | ** |
| Abnormal blood chloride concentration | 3.86 | [3.35~4.44] | 2.31 x 10^-80^ | ** | 0.72 | [0.66~0.79] | 1.83 x 10^-12^ | ** |
| Abnormal blood inorganic cation concentration | 3.18 | [2.78~3.64] | 7.69 x 10^-65^ | ** | 0.72 | [0.66~0.78] | 5.32 x 10^-14^ | ** |
| Abnormal circulating creatinine level | 2.73 | [2.39~3.12] | 8.95 x 10^-50^ | ** | 0.72 | [0.66~0.78] | 6.22 x 10^-14^ | ** |
| Decreased glomerular filtration rate | 3.72 | [3.29~4.2] | 1.50 x 10^-99^ | ** | 0.72 | [0.66~0.8] | 1.02 x 10^-10^ | ** |
| Elevated gamma-glutamyltransferase activity | 6.39 | [5.42~7.52] | 3.34 x 10^-111^ | ** | 0.72 | [0.61~0.86] | 2.83 x 10^-4^ | ** |
| Elevated serum creatinine | 2.5 | [2.22~2.83] | 1.25 x 10^-50^ | ** | 0.72 | [0.66~0.79] | 1.75 x 10^-12^ | ** |
| Hyperchloremia | 3.65 | [3.21~4.16] | 6.26 x 10^-88^ | ** | 0.72 | [0.65~0.79] | 6.38 x 10^-11^ | ** |
| Hypomagnesemia | 3.9 | [3.41~4.46] | 8.05 x 10^-90^ | ** | 0.72 | [0.64~0.82] | 1.94 x 10^-7^ | ** |
| Abnormal blood sodium concentration | 2.93 | [2.57~3.34] | 7.43 x 10^-59^ | ** | 0.71 | [0.64~0.78] | 2.27 x 10^-12^ | ** |
| Abnormal circulating nitrogen compound concentration | 2.99 | [2.55~3.51] | 1.18 x 10^-41^ | ** | 0.71 | [0.65~0.77] | 2.51 x 10^-14^ | ** |
| Abnormal homeostasis | 2.57 | [2.16~3.06] | 7.38 x 10^-27^ | ** | 0.71 | [0.64~0.78] | 1.47 x 10^-12^ | ** |
| Abnormal magnesium concentration | 4.04 | [3.56~4.59] | 7.57 x 10^-105^ | ** | 0.71 | [0.64~0.79] | 7.01 x 10^-11^ | ** |
| Alkalemia | 2.75 | [2.18~3.48] | 1.19 x 10^-17^ | ** | 0.71 | [0.57~0.9] | 3.38 x 10^-3^ | ** |
| Hyponatremia | 2.2 | [1.93~2.5] | 5.17 x 10^-33^ | ** | 0.71 | [0.64~0.79] | 6.14 x 10^-11^ | ** |
| Abnormal circulating calcium concentration | 2.74 | [2.41~3.12] | 1.04 x 10^-54^ | ** | 0.7 | [0.64~0.76] | 1.41 x 10^-15^ | ** |
| Abnormal HDL cholesterol concentration | 2.92 | [2.53~3.36] | 3.17 x 10^-51^ | ** | 0.7 | [0.63~0.79] | 8.17 x 10^-10^ | ** |
| Abnormal red blood cell count | 3.67 | [3.16~4.27] | 3.98 x 10^-66^ | ** | 0.7 | [0.64~0.77] | 1.21 x 10^-15^ | ** |
| Hypermagnesemia | 4.03 | [3.5~4.65] | 3.26 x 10^-85^ | ** | 0.7 | [0.61~0.8] | 7.63 x 10^-8^ | ** |
| Increased serum ferritin | 4.36 | [3.3~5.77] | 1.85 x 10^-25^ | ** | 0.7 | [0.52~0.95] | 1.90 x 10^-2^ | * |
| Reduced hematocrit | 3.76 | [3.25~4.35] | 7.67 x 10^-73^ | ** | 0.7 | [0.64~0.76] | 4.67 x 10^-17^ | ** |
| Abnormal circulating protein level | 5.6 | [4.83~6.5] | 4.93 x 10^-117^ | ** | 0.69 | [0.63~0.76] | 1.39 x 10^-15^ | ** |
| Hypocalcemia | 2.54 | [2.25~2.88] | 4.74 x 10^-50^ | ** | 0.69 | [0.63~0.76] | 4.81 x 10^-16^ | ** |
| Hypoproteinemia | 3.48 | [3.05~3.97] | 3.54 x 10^-79^ | ** | 0.69 | [0.62~0.77] | 1.32 x 10^-11^ | ** |
| Abnormal lymphocyte count | 5.53 | [4.79~6.38] | 9.71 x 10^-124^ | ** | 0.68 | [0.61~0.74] | 3.53 x 10^-16^ | ** |
| Abnormal lymphocyte morphology | 5.53 | [4.79~6.38] | 9.71 x 10^-124^ | ** | 0.68 | [0.61~0.74] | 3.53 x 10^-16^ | ** |
| Abnormal serum ferritin | 4.01 | [3.11~5.18] | 3.85 x 10^-27^ | ** | 0.68 | [0.52~0.9] | 5.18 x 10^-03^ | ** |
| Abnormal blood urea nitrogen concentration | 2.5 | [2.2~2.85] | 2.78 x 10^-44^ | ** | 0.67 | [0.62~0.74] | 1.15 x 10^-18^ | ** |
| Azotemia | 2.43 | [2.13~2.76] | 3.48 x 10^-42^ | ** | 0.66 | [0.6~0.72] | 6.81 x 10^-22^ | ** |
| Hyperkalemia | 4.25 | [3.7~4.87] | 2.44 x 10^-96^ | ** | 0.66 | [0.58~0.75] | 8.07 x 10^-11^ | ** |
| Increased mean platelet volume | 3.5 | [3.01~4.07] | 4.35 x 10^-61^ | ** | 0.66 | [0.57~0.76] | 8.72 x 10^-9^ | ** |
| Increased mean corpuscular volume | 1.82 | [1.57~2.11] | 1.72 x 10^-15^ | ** | 0.65 | [0.57~0.74] | 3.75 x 10^-11^ | ** |
| Leukopenia | 5.21 | [4.52~6.01] | 2.99 x 10^-117^ | ** | 0.65 | [0.6~0.72] | 1.48 x 10^-19^ | ** |
| Abnormality of metabolism/homeostasis | 2.71 | [1.69~4.33] | 2.66 x 10^-0]5^ | ** | 0.64 | [0.52~0.79] | 2.23 x 10^-5^ | ** |
| Conjugated hyperbilirubinemia | 2.53 | [1.99~3.22] | 1.63 x 10^-14^ | ** | 0.64 | [0.5~0.81] | 1.70 x 10^-4^ | ** |
| Hypoglycemia | 3.59 | [2.97~4.35] | 7.42 x 10^-40^ | ** | 0.64 | [0.52~0.77] | 5.73 x 10^-6^ | ** |
| Lymphopenia | 5.45 | [4.74~6.26] | 6.89 x 10^-129^ | ** | 0.64 | [0.58~0.7] | 3.46 x 10^-20^ | ** |
| Neutropenia | 3.24 | [2.79~3.77] | 3.29 x 10^-54^ | ** | 0.64 | [0.55~0.74] | 8.93 x 10^-10^ | ** |
| Prolonged partial thromboplastin time | 3.43 | [2.97~3.96] | 1.01 x 10^-64^ | ** | 0.64 | [0.56~0.73] | 3.44 x 10^-11^ | ** |
| Decreased red blood cell count | 3.34 | [2.92~3.83] | 7.20 x 10^-69^ | ** | 0.63 | [0.58~0.69] | 1.22 x 10^-26^ | ** |
| Abnormal platelet count | 2.58 | [2.28~2.92] | 4.91 x 10^-52^ | ** | 0.62 | [0.57~0.69] | 1.99 x 10^-22^ | ** |
| Acellular urinary casts | 5.39 | [4.44~6.55] | 1.00 x 10^-65^ | ** | 0.62 | [0.5~0.76] | 6.90 x 10^-6^ | ** |
| Cylindruria | 5.39 | [4.44~6.55] | 1.00 x 10^-65^ | ** | 0.62 | [0.5~0.76] | 6.90 x 10^-6^ | ** |
| Hyaline casts | 5.39 | [4.44~6.55] | 1.00 x 10^-65^ | ** | 0.62 | [0.5~0.76] | 6.90 x 10^-6^ | ** |
| Hypophosphatemia | 4.08 | [3.44~4.84] | 3.74 x 10^-59^ | ** | 0.61 | [0.51~0.73] | 3.89 x 10^-8^ | ** |
| Hyperbilirubinemia | 2.26 | [1.92~2.66] | 5.70 x 10^-23^ | ** | 0.59 | [0.51~0.69] | 3.94 x 10^-12^ | ** |
| Increased blood urea nitrogen | 2.08 | [1.84~2.36] | 1.74 x 10^-32^ | ** | 0.59 | [0.54~0.64] | 4.39 x 10^-31^ | ** |
| Abnormal albumin level | 10.59 | [9.03~12.43] | 5.94 x 10^-188^ | ** | 0.58 | [0.48~0.7] | 4.26 x 10^-9^ | ** |
| Abnormal circulating metabolite concentration | 4.25 | [2.83~6.39] | 1.96 x 10^-12^ | ** | 0.58 | [0.5~0.68] | 2.94 x 10^-12^ | ** |
| Abnormality of coagulation | 3.31 | [2.92~3.76] | 2.04 x 10^-78^ | ** | 0.58 | [0.53~0.65] | 8.21 x 10^-25^ | ** |
| Abnormality of the coagulation cascade | 3.36 | [2.95~3.83] | 2.14 x 10^-75^ | ** | 0.57 | [0.51~0.64] | 7.89 x 10^-23^ | ** |
| Hypoalbuminemia | 10.41 | [8.86~12.22] | 4.41 x 10^-183^ | ** | 0.57 | [0.48~0.69] | 3.31 x 10^-9^ | ** |
| Abnormal circulating nucleobase concentration | 6.89 | [5.62~8.45] | 1.16 x 10^-78^ | ** | 0.56 | [0.44~0.71] | 1.52 x 10^-6^ | ** |
| Abnormal circulating purine concentration | 6.89 | [5.62~8.45] | 1.16 x 10^-78^ | ** | 0.56 | [0.44~0.71] | 1.52 x 10^-6^ | ** |
| Abnormal prothrombin time | 3.34 | [2.93~3.8] | 3.64 x 10^-74^ | ** | 0.55 | [0.49~0.62] | 5.10 x 10^-24^ | ** |
| Abnormality of prothrombin | 3.34 | [2.93~3.8] | 3.64 x 10^-74^ | ** | 0.55 | [0.49~0.62] | 5.10 x 10^-24^ | ** |
| Prolonged prothrombin time | 3.2 | [2.81~3.65] | 7.49 x 10^-69^ | ** | 0.55 | [0.49~0.61] | 1.07 x 10^-24^ | ** |
| Abnormal circulating beta globulin level | 8.29 | [6.46~10.64] | 1.83 x 10^-63^ | ** | 0.54 | [0.39~0.73] | 5.58 x 10^-5^ | ** |
| Abnormal circulating globulin level | 8.29 | [6.46~10.64] | 1.83 x 10^-63^ | ** | 0.54 | [0.39~0.73] | 5.58 x 10^-5^ | ** |
| Increased total bilirubin | 2.23 | [1.87~2.66] | 1.79 x 10^-19^ | ** | 0.54 | [0.45~0.64] | 3.08 x 10^-13^ | ** |
| Thrombocytopenia | 2.03 | [1.79~2.31] | 1.37 x 10^-27^ | ** | 0.53 | [0.48~0.59] | 7.22 x 10^-31^ | ** |

asterisk: p < 0.05, double asterisk: p < 0.01; table is sorted by the odds ratio for acute asthma diagnosis
